# Vitrectomy with sulfur hexafluoride versus air tamponade for idiopathic macular hole: a retrospective study

**DOI:** 10.1186/s12886-023-03049-2

**Published:** 2023-07-20

**Authors:** Yuou Yao, Huichao Yan, Jinfeng Qu, Chongya Dong, Jianhong Liang, Hong Yin, Chi Ren, Enzhong Jin, Mingwei Zhao

**Affiliations:** 1 Department of Ophthalmology, Peking University People’s Hospital, Xizhimen South Street 11, Xi Cheng District, Beijing, 100044 China; 2 Eye Diseases and Optometry Institute, Beijing, China; 3 Beijing Key Laboratory of Diagnosis and Therapy of Retinal and Choroid Diseases, Beijing, China; 4 College of Optometry, Peking University Health Science Center, Beijing, China; 5 Department of Biostatistics, Peking University Clinical Research Institute, Beijing, China

**Keywords:** Tamponade, Gas, Retinal perforations, Surgery

## Abstract

**Background:**

To evaluate the effect of room air and sulfur hexafluoride (SF6) gas in idiopathic macular hole(MH)surgery.

**Methods:**

Retrospective, interventional, and comparative study. 238 eyes with the idiopathic macular hole that underwent pars plana vitrectomy, internal limiting membrane peeling, fluid-air exchange, and 20% SF6 (SF6 group:125 eyes) or room air tamponade (air group: 113 eyes) were reviewed. The primary outcome measure was the closure rate of primary surgery.

**Results:**

The baseline characteristics of the SF6 group and air group were comparable except for the hole size (479.90 ± 204.48 vs. 429.38 ± 174.63 μm, *P* = 0.043). The anatomical closure rate was 92.8% (116 / 125) with the SF6 group and 76.1% (86 / 113) with the air group (*P* < 0.001). A cut-off value of MH size to predict primary anatomical closure was 520 μm, which is based on the lower limit of 95% confidential interval of the MH size among the unclosed patients in the air group. There was no significant difference in anatomical closure rates between SF6 and air group (98.7% vs. 91.9%, *P* = 0.051) for MH ≤ 520 μm, whereas a significantly lower anatomical closure rate was shown in the air group than SF6 group (46.2% vs. 84.0%, *P* < 0.001) for MH > 520 μm.

**Conclusion:**

SF6 exhibited more effectiveness than air to achieve a good anatomical outcome for its longer tamponade when MH > 520 μm.

**Supplementary Information:**

The online version contains supplementary material available at 10.1186/s12886-023-03049-2.

## Background

Nowadays, the standard treatment for full-thickness macular hole (FTMH) is pars plana vitrectomy (PPV), internal limiting membrane (ILM) peeling, gas tamponade, and when the macular hole size is larger than 400 μm, face down position is significant for higher closure rate [1–4]. As for the gas tamponade, more surgeons prefer to use sulfur hexafluoride (SF6) because it can not only achieve a similar success rate as perfluoro ethane (C2F6) and perfluoro propane (C3F8) but also reduced the negative impact on patient’s daily activity and related complications [5–7]. Recently, several studies have concluded that air provided equivalent MH closure rates compared to SF6 [8, 9], and had shorter tamponade time. However, their conclusion was limited by relatively small sample size (22 patients) [8], small hole size (mean MH size ≤ 400 μm) [5, 9], or variable surgical techniques which may affect the validation and application of the results. Our study is a more strictly designed study that aims to compare the anatomical and functional outcomes of vitrectomy with SF6 or air tamponade for idiopathic macular holes, especially in large diameters of macular holes. And try to find out the cut-point of the MH size for different gas tamponade.

## Method

### Study design

The study adhered to the Declaration of Helsinki and was approved by the Peking University People’s Hospital research ethics committees and the Peking University institutional review board. This is a observational, retrospective, interventional, comparative study of idiopathic MH patients whose data were collected from two prospective studies of our group which shared the same protocol except for the gas tamponade (NCT02930369, NCT 02905409). The study adhered to the Declaration of Helsinki and was approved by the Peking University People’s Hospital research ethics committees and the Peking University institutional review board.

### Patient selection

Treatment-naive full-thickness idiopathic MH patients who underwent PPV in Peking University People’s Hospital from May 2012 to June 2019 were selected by our study. The inclusion criteria included: [1] less than or equal to 3 years duration (based on symptoms reported by the patient) [2]. the surgical procedure that had been standardized in our previous studies (NCT02930369, NCT 02905409), including standard 23- or 25-gauge pars plana vitrectomy with indocyanine green-assisted ILM peeling and 20% SF6 or filtered air tamponade combined with or without phacoemulsification and intraocular lens implantation [3]. a minimum follow-up of 6 months. The exclusion criteria included: [1] high myopia (> 6 diopters) [2]. macular hole was secondary to other fundus diseases [3]. the presence of other ocular diseases which may cause decreased vision [4]. retinal detachment due to macular hole [5]. history of previous vitrectomy. For patients with bilateral MHs eligible, only the eye which underwent PPV first was enrolled. The study protocol was approved by the institutional ethics committee.

Gas tamponade was 20% SF6 for idiopathic MH patients in a previous study (NCT02930369), and thus was analyzed as the SF6 group. Phacoemulsification and lens implantation was performed if a cataract was present in the SF6 group unless pseudophakic eye. And the other study (NCT02905409) followed the surgical protocol of air tamponade, which was analyzed as the air group. All patients in the air group underwent phacoemulsification and lens implantation unless they were already pseudophakic before the surgery. Patients were instructed to maintain a prone position until the gas bubble was absorbed absolutely when the MH size was larger than 400 μm, and other patients were instructed to maintain a prone position as the control variable.

### Data collection

Data obtained for each patient included age, gender, duration of symptoms, peeling area, macular hole size, and lens status (phakic, pseudophakic, or aphakic) at baseline and best corrected visual acuity (BCVA) measured by Early Treatment Diabetic Retinopathy Study (ETDRS) chart at 4 m, intraocular pressure measurement, slit-lamp examination of the anterior segment, dilated fundus examination and spectral domain optical coherence tomography [10] (SD-OCT, Optovue, Fremont, CA, US, Heidelberg Engineering, Heidelberg, Germany) at baseline and each postoperative visit. Hole sizes were defined as the shortest distances between the edges of the broken ends of the detached neurosensory retina in the OCT B-scan with the maximum dimensions.

The primary outcome was the hole closure rate of the primary surgery. The second outcome was the proportion of the eyes that BCVA improved at least 10 ETDRS letters at 6 months. Patients with MH unclosed at the first postoperative visit within 1 month were considered as surgical failure, and were recommended to receive reoperation.

### Statistical analysis

In the univariate analyses, PASS 2019 (PASS for Windows, Kaysville,USA) was used to calculate power, the significance level of the test is 0.05, continuous variables were compared using an independent sample two-tailed Student’s t-test. And chi-square test was conducted in subgroup analysis to compare closure rates in 2 groups with different hole sizes. We defined the cut-off value based on the lower limit of 95% CI of the MH size among the unclosed patients both in the air group and SF6 group. Binary logistic regression analyses were used to analyze the effect of single parameters on closure rate in different size macular holes. In addition to p-values for the influence as predictors for the closure of the macular hole, Odds Ratios (ORs) were calculated to estimate the strength of influence, each with a 95% confidential interval (CI). And risk factors for the primary anatomical failure of MH surgery between the SF6 group and the air group were performed using the Mann-Whitney U test. SPSS 26.0 (SPSS for Windows, Chicago, IL) was used in all the statistical analyses of this study. A *P* value of 0.05 or less was considered statistically significant.

## Results

### Baseline demographic characteristics

A total of 238 eyes from 238 patients were included in this study, of which 125 eyes were in the SF6 group and 113 eyes were in the air group. Group sample size of 125 in SF6 group and 113 in air group achieved power of 95.02%. The demographic and characteristics of all patients and patients in each group are shown in Table 1. All baseline characteristics between the SF6 group and air group were comparable, except for the hole size (479.90 ± 204.48 μm vs. 429.38 ± 174.63 μm, *P* = 0.043, independent sample t-test). The average peeling range of all eyes during the operation is (2.96 ± 0.99)×(2.95 ± 0.98) papillary diameter(PD). One eye in the SF6 group and 4 eyes in the air group were pseudophakic preoperatively. The mean follow-up of all patients was 10.2 ± 3.2 months.

**Table 1:** Patients’ characteristics

	Total	Group		
		SF6	Air	*P* value
Age(yrs)	64.50 ± 6.44	64.54 ± 6.65	64.46 ± 6.24	0.928*
Gender (male/female, n)	58/180	32/93	26/87	0.642†
Laterality (od/os)	109/129	56/69	53/60	0.745†
Duration of Symptoms (mons)	6.11 ± 10.45	5.04 ± 9.55	7.30 ± 11.30	0.095*
MH size (µm)	455.92 ± 192.16	479.90 ± 204.48	429.38 ± 174.63	0.043*
Preop BCVA (ETDRS letters)	41.41 ± 15.23	41.85 ± 15.49	40.92 ± 14.98	0.639*
Combined surgery(n,%)	214(89.9%)	105(84.0%)	109(96.5%)	< 0.001†

*Independent sample test. †Pearson chi-square test. ‡ Fisher’s exact test. §Mann–Whitney U test. BCVA best corrected visual acuity, ETDRS Early Treatment Diabetic Retinopathy Study

### The cut-off value of MH size for different gas

**Fig. 1:**
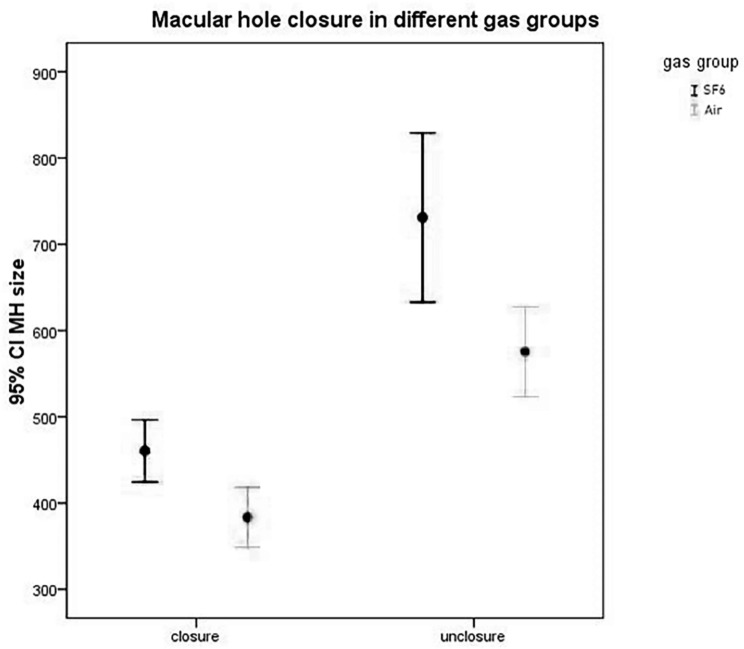
Macular hole closure in different gas groups, SPSS, Error Bar Chart

The average MH size of closed patients in the air group was 383.55 ± 17.38 μm (95%CI 348.99–418.10 μm), and the average MH size of unclosed patients was 575.37 ± 25.36 μm (95%CI 523.25–627.49 μm). Whereas in the SF6 group, the mean MH size of closed and unclosed patients was 460.42 ± 18.25 μm (95% CI 424.27–496.57 μm), and 731.00 ± 42.58 μm (95%CI 632.81-829.19 μm), respectively.(Figure 1).

Accordingly, we defined 520 μm as the cut-off value based on the lower limit of 95% CI of the MH size among the unclosed patients in the air group. Similarly, we defined 630 μm as the cut-off value in the SF6 group.

### Anatomical outcome and correlated factors

Stratified the patients based on the MH size of 520 μm, the primary MH closure rates of the SF6 group and air group were shown in Table 2. In the large MH size subgroup (> 520 μm), the MH closure rate of the air group was 46.2% (18/38), which was much lower than that of the SF6 group 84.0% (42/50), showed a significant difference (*P* < 0.001, Pearson chi-square test). In the small MH size group (≤ 520 μm), the MH closure rate of the air and SF6 group was similar (91.9% vs. 98.7%, respectively, P = 0.051, Fisher’s exact test).

**Table 2:** The anatomical outcome of 2 groups stratified by MH size

	MH size ≤ 520 μm			MH size > 520 μm			*P* value
	success	failed	*P* value	success	failed	*P* value	
SF6 group (n, %)	74(98.7%)	1(1.3%)	0.051‡	42(84.0%)	8(16.0%)	< 0.001†	0.002‡
Air group (n, %)	68(91.9%)	6(8.1%)		18(46.2%)	21(53.8%)		< 0.001†

*Independent sample test. †Pearson chi-square test. ‡ Fisher’s exact test.

The primary hole closure rate was 84.9% (202/238) in total, 92.8% (116/125) in the SF6 group, and 76.1% (86/113) in the air group. The closure rate of the air group was significantly lower than that of the SF6 group (*P* < 0.001, Pearson chi-square test). In the SF6 group, eyes with MH size ≤ 520 μm showed a significantly higher closure rate than MH size > 520 μm (98.7% vs. 84.0%, *P* = 0.002, Fisher’s exact test), and this difference in the air group was more remarkable (91.9% vs. 46.2%, *P* < 0.001, Pearson chi-square test).

According to the results of binary logistic regression analysis shown in Table 3, when the macular hole size ≤ 520 μm, the age and the type of gas does not affect the closure rate. (P > 0.05), while the duration of the disease can affect the closure rate. (P = 0.03). However, when the hole size>520 μm, the age and the type of gas can affect the closure rate (P < 0.01), as well as the duration of disease (P = 0.01).

**Table 3:** Binary logistic regression analysis of macular hole healing rate

Size	Parameter	P	OR	95%CI
≤ 520 μm	Gas Type	0.09	7.31	0.736–72.60
	Age	0.63	0.97	0.84–1.11
	Duration of Disease	0.03	1.05	1.01–1.10
>520 μm	Gas Type	<0.01	6.23	2.01–19.32
	Age	<0.01	1.18	1.06–1.32
	Duration of Disease	0.01	1.07	1.02–1.13

The 36 patients who failed to achieve MH closure in primary PPV had an average hole size of 614.28 ± 145.93 μm. The baseline characteristics of the patients who failed to achieve MH closure in both groups were comparable except for the MH size, which was significantly larger in the SF6 group (731.00 ± 127.74 vs. 575.37 ± 131.75 μm, *P* = 0.004). (Table 4)

**Table 4:** Details of primary failed MH in both groups

	SF6 Group	Air Group	*P* value
No of eyes (n)	9	27	
Age(yrs)	68.22 ± 6.22	67.11.92 ± 5.56	0.464§
Duration(mons)	24.39 ± 24.88	10.55 ± 12.60	0.061§
MH size(µm)	731.00 ± 127.74	575.37 ± 131.75	0.004§
Baseline BCVA (ETDRS letters)	38.11 ± 16.20	33.85 ± 13.27	0.509§
Final BCVA (ETDRS letters)	48.44 ± 14.33	39.26 ± 12.40	0.087§

§Mann–Whitney U test.

### Functional outcome

The mean BCVA of all patients at 6 months visit was 63.94 ± 13.16 ETDRS letters, and significantly improved by an average of 21.66 ± 16.15 ETDRS letters from baseline (*P* < 0.001, paired t-test). The proportion of eyes with BCVA improved more than 2 lines was 78.9% (172 / 218) in total. The mean BCVA improvement after the surgery in the SF6 group was significantly higher than in the air group (24.40 ± 16.47 vs. 18.24 ± 15.13 ETDRS letters, *P* = 0.005, independent t-test). However, when analyzed patients achieved primary hole closure, improvement of BCVA was comparable between the SF6 and the air group (25.52 ± 16.11 vs. 21.89 ± 13.54 ETDRS letters, *P* = 0.109, independent t-test). The proportion of eyes with BCVA improved more than 2 lines was similar in both groups (87.6% vs. 84.9%, *P* = 0.601, Pearson chi-square test).

## Discussion

The expected duration of the gas bubble for MH closure has no consensus, leading to the surgeon’s discretion in the choice of gas type. Recently, surgeons were inclined to use shorter-lasting gas such as SF6 [11] which can provide similar surgical outcomes, lower incidence of gas-related adverse events, and shorter disturbance of daily life compared with C2F6 and C3F8, irrespective of stage, size, or duration of MHs [12–15]. Based on the evidence that hole closure occurs often within the first postoperative 24 h observed on OCT [16, 17], sterilized air is expected to replace SF6 or other longer-lasting gas since it is the known gas with the shortest intraocular lasting period. We performed the present study to find out the effectiveness of air in MH surgery and find out that air might provide similar effectiveness as SF6 for patients with MH size smaller than 520µmwhich is different from most previous studies that used 400 μm as the cut-off point of large MH based on Gass’s staging system [18] or international vitreomacular traction study group suggested in 2013 [19] It is identified that air is effective for small MH. Usui et al. [8] retrospectively studied patients with an average of 303 μm and 227 μm in the SF6 group and air group respectively, for whom achieved a 100% closure rate, and Tao et al. [20] confirmed that with an average MH size of 255 μm. Hasegawa et al. [9] included patients with a mean hole diameter of 352 μm in the SF6 group and 370 μm in the air group, who achieved a similar closure rate of around 91.0%. Recently, there is a multicenter, randomized controlled, non-inferiority study suggesting that air tamponade is inferior to SF_6_ tamponade for MHs of ≤ 400 μm in diameter [21], which is in contrast to our result. However, there are certain differences between the two studies in the sample size and baseline data including age, course of disease, and so on, which may lead to different conclusions. What’s more, the p-value in our study is close to 0.05 (P = 0.051), but the macular hole closure rate in the air group is a little bit lower than that in the SF6 group (91.9% vs. 98.7%), which may have clinical significance.

However, for large diameter macular holes (> 400 μm), the MH size boundary of short-term effect gas tamponades such as SF6 and air are controversial. Many researchers reported different cut-off points of large MH in certain circumstances recently. Steel et al. [22] found a cut-off of 500 μm as a new pragmatic size definition of large MHs for the surgical treatment using various long-lasting gas tamponade including SF6, C2F6, C3F8, and various ILM peeling techniques. The present study was based on the data of 2 well-designed prospective clinical trials sharing the same protocol except for the gas type. And the mean MH size was 455.92 μm in the present study, which was much larger than previous literature concerning the air tamponade, filling the gap in this field [22–24]. MHs > 520 μm achieved an anatomical closure rate of 46.2%, in contrast with the high closure rate of 91.9% in MHs ≤ 520 μm in the air group, which may indicate that air has a good effect on macular holes when MHs ≤ 520 μm, expanded the indication of air tamponade use for MH size from 400 μm in previous studies to 520 μm. However, few surgeons preferred to use air in the real world. Jackson et al. [14] reported 2.2% and Steel et al. [11] reported only 0.3% of air tamponade used in 2 studies involving a large cohort of more than 1000 patients. By providing the validated evidence for surgeons to choose the gas tamponade during the MH surgery, we hope to change the current situation.

In this study, the postoperative BCVA of both groups improved significantly compared with preoperative BCVA, and the SF6 group improved greater than the air group. However, taking the lower anatomical closure rate of the air group into consideration, patients who achieved primary anatomical closure of 2 groups showed similar BCVA improvement (P = 0.120, Mann–Whitney U test). Furthermore, because our previous study [25] proved that postoperative BCVA was significantly correlated with anatomical outcomes, primary surgical success should be considered as the primary goal of the surgery.

The present study indicated that patients with longer duration, larger MH, and elder age were vulnerable to experiencing surgical failure, which is consistent with many previous studies [9, 11, 26]. And for patients with primary failed surgical outcome, the MH size of the air group was smaller than SF6 group (587.38 ± 122.17 μm vs. 684.0 ± 91.56 μm, respectively), which shows a significant difference for the small sample (*P* = 0.049, Mann–Whitney U test), providing another evidence that SF6 might be more effective for large MHs than air does. Furthermore, it should be noted that our previous study and other studies have shown that the gauge size does not affect the closure rate [25, 27].

The present study did not analyze the adverse events corresponding to tamponade agents of the 2 groups. However, many studies [12, 15] have already elucidated that SF6 has a lower incidence of glaucoma, cataract progression, and pupillary capture than C3F8 and C2F6.

Limitations of this study include its retrospective design and lack of adverse event data. Thus, a well-designed, adequately powered, prospective, randomized, controlled clinical trial concerning the expansile gas and air tamponade effect should be conducted to replicate our results and to determine with confidence its value. What’s more, our study is a retrospective study, and its sample size is based on the previous collected database. The sample size of this study may affect the analysis especially the subgroup analysis, thus the further prospective study is needed in the future.

In conclusion, what’s more important, for patients with large MH, like MH size > 520 μm, SF6 tamponade is more effective than air to achieve good anatomical and functional outcomes for its longer tamponade. Air may provide similar effect with MH ≤ 520 μm to achieve hole closure and BCVA improvement as SF6, and further research is needed to validate the effectiveness of air and SF6 on the closure rate of small-size macular holes.

## Electronic supplementary material

Below is the link to the electronic supplementary material.

Supplementary Material 1

## Data Availability

All data generated or analyzed during this study are included in this published article.

## Abbreviations

BCVA: Best corrected visual acuity
C2F6: Perfluoro ethane
C3F8: Perfluoro propane
ETDRS: Early Treatment Diabetic Retinopathy Study
FTMH: Full-thickness macular hole
ILM: Internal limiting membrane
MH: Macular hole
PD: Papillary diameter
PPV: Pars plana vitrectomy
SD-OCT: Spectral domain optical coherence tomography
SF6: Sulfur hexafluoride
